# Computational Network Analysis Identifies Evolutionarily Conserved miRNA Gene Interactions Potentially Regulating Immune Response in Bovine Trypanosomosis

**DOI:** 10.3389/fmicb.2019.02010

**Published:** 2019-08-28

**Authors:** Olanrewaju B. Morenikeji, Megan E. Hawkes, André O. Hudson, Bolaji N. Thomas

**Affiliations:** ^1^Department of Biomedical Sciences, Rochester Institute of Technology, Rochester, NY, United States; ^2^Thomas H. Gosnell School of Life Sciences, Rochester Institute of Technology, Rochester, NY, United States

**Keywords:** cattle, miRNA-target, expression, trypanosomosis, evolution, immune response

## Abstract

Bovine trypanosomosis is a devastating disease that causes huge economic loss to the global cattle industry on a yearly basis. Selection of accurate biomarkers are important in early disease diagnosis and treatment. Of late, micro-RNAs (miRNAs) are becoming the most useful biomarkers for both infectious and non-infectious diseases in humans, but this is not the case in animals. miRNAs are non-coding RNAs that regulate gene expression through binding to the 3′-, 5′-untranslated regions (UTR) or coding sequence (CDS) region of one or more target genes. The molecular identification of miRNAs that regulates the expression of immune genes responding to bovine trypanosomosis is poorly defined, as is the possibility that these miRNAs could serve as potential biomarkers for disease diagnosis and treatment currently unknown. To this end, we utilized *in silico* tools to elucidate conserved miRNAs regulating immune response genes during infection, in addition to cataloging significant genes. Based on the *p* value of 1.77E-32, we selected 25 significantly expressed immune genes. Using prediction analysis, we identified a total of 4,251 bovine miRNAs targeting these selected genes across the 3′UTR, 5′UTR and CDS regions. Thereafter, we identified candidate miRNAs based on the number of gene targets and their abundance at the three regions. In all, we found the top 13 miRNAs that are significantly conserved targeting 7 innate immune response genes, including bta-mir-2460, bta-mir-193a, bta-mir-2316, and bta-mir-2456. Our gene ontology analysis suggests that these miRNAs are involved in gene silencing, cellular protein modification process, RNA-induced silencing complex, regulation of humoral immune response mediated by circulating immunoglobulin and negative regulation of chronic inflammatory response, among others. In conclusion, this study identifies specific miRNAs that may be involved in the regulation of gene expression during bovine trypanosomosis. These miRNAs have the potential to be used as biomarkers in the animal and veterinary research community to facilitate the development of tools for early disease diagnosis/detection, drug targeting, and the rational design of drugs to facilitate disease treatment.

## Introduction

Every year, the Cattle industry is faced with huge economic losses due to diseases such as trypanosomosis. Bovine trypanosomosis is endemic in sub-Saharan Africa and some parts of The Americas, causing losses of several billion dollars annually (Abdi et al., 2017; Odeniran and Ademola, 2018). The disease is associated with multifactorial complex phenotype, requiring an integrative biological approach to elucidate the molecular networks involved and to identify significant markers that may be useful for disease diagnosis and treatment (Noyes et al., 2011; Smetko et al., 2015; Kim et al., 2017). Recently, major research efforts are focused on utilizing microRNA (miRNA) as diagnostic biomarkers or therapeutic targets due to their involvement in various disease phenotypes and malignancies (Lawless et al., 2014; Scheel et al., 2017; Ammah et al., 2018; da Silveira et al., 2018). MicroRNAs are short, non-coding RNAs, which are capable of post-transcriptionally modifying gene expression, and form complexes with argonaute proteins that hybridize to the target mRNAs leading to the degradation of mRNA and negatively impact protein expression (Bushati and Cohen, 2007; Bartel, 2009; Meister, 2013; Huberdeau et al., 2017).

Post-transcriptional modification of gene expression is becoming a major focus in disease studies (Liu and Wang, 2019), with several reports showing miRNAs as important players in post-transcriptional regulation of both innate and adaptive immune gene expression (O’Connell et al., 2010; Sandhu et al., 2012; Kramer et al., 2015; Mehta and Baltimore, 2016; Pichulik et al., 2016; Kumar et al., 2018; Awuah et al., 2019). miRNAs have also been implicated in the regulation of immune functions in different cell types, for example neutrophil senescence and naïve mouse B cells have been reported to be regulated by repertoires of miRNAs (Ward et al., 2011; Bronevetsky et al., 2013; Aalaei-Andabili and Rezaei, 2016; Kangas et al., 2017). While there are numerous miRNAs abundantly expressed in bovine tissues and the entire genome, there are very few studies elucidating their regulatory role in bovine immunity (Coutinho et al., 2007; Jin et al., 2009; Xu et al., 2009; Hanif et al., 2018; Li et al., 2019). MicroRNAs therefore, will play important and unique roles during immune response, including modification of gene expression during bovine trypanosomosis. For instance, mir-2284 and mir-2285 families have been associated with TLR genes, thereby regulating non-specific immune responses through the production of pro-inflammatory cytokines, suggesting a role in bovine disease tolerance (Das et al., 2016). Similarly, mir-2404, mir-2285, and mir-6522 have been reported to regulate CD8 alpha chain and T-cell surface glycoprotein, thus playing significant role in development of T and B cell, proliferation, and inflammatory process (Mahjoub et al., 2015; Hanif et al., 2018). Studies have shown that miRNAs repress the target genes through the 3′ untranslated region (3′UTR) of mRNAs, therefore the miRNA binding site in this region seems to be well characterized (Buza et al., 2014; Liu and Wang, 2019). However, comparative genome analysis has shown that both coding domain sequence (CDS) and 3′UTR sites are under selective pressure while many miRNA binding sites are conserved in both 3′UTR and CDS (Hausser et al., 2013; Brummer and Hausser, 2014). In fact, many investigators are suggesting an alternative mode of gene regulation, whereby miRNAs anneal within the CDS, 5′- and/or 3′-UTR regions of their targets thereby regulating their translation.

Hence, there remains an important need to identify possible miRNA binding sites within the complete sequence of a gene (i.e., 5′-UTR, CDS and 3′-UTR), which can serve as potential biomarkers for disease diagnosis and treatment. To date, there has not been a published study identifying potential miRNAs as biomarkers in the entire sequence of immune genes responding during bovine trypanosomosis, demonstrating the uniqueness of this study. Notably, there are many miRNA databases and algorithms, which are available to investigate significant pathways and transcriptional mechanisms within various interaction and expression datasets (Xie et al., 2013; Li et al., 2014; Hanif et al., 2018). There are also proven *in silico* tools and algorithms available for miRNA target prediction and for dissecting miRNA function (Kogelman et al., 2014; Scheel et al., 2017; Hanif et al., 2018). A quantitative measurement of miRNA expression in conjunction with their target gene expression would be an important means to deconvolute diverse biogenesis pathways and immune system regulation during bovine trypanosomosis. To this end, we have employed a combination of computational tools to identify and elucidate conserved miRNAs, their target interaction(s), and regulatory function(s) during bovine trypanosomosis. This study, we believe, is a proof of concept with the potential to guide future studies aiming to identify attractive and putative miRNA targets for the rationale design and/or discovery of drugs to combat diseases, including potential biomarkers for disease diagnosis.

## Materials and Methods

### Data Mining and Verification of Genes Associated With Bovine Trypanosomosis Pathway

In this study, we performed a data survey of genes that are reported to be significantly regulated during bovine trypanosomosis through publicly available databases. Briefly, we used LitInspector of Genomatix Literature Mining software (Version 3.10, Munich, Germany^1^) that uses proprietary literature data mining algorithms based on all available PubMed publications and their corresponding Medical Subject Headings (MeSH) (Berriz et al., 2003; Maier et al., 2005). This program gathers gene information and correlations with diseases and pathways from published literature. The gene sets are characterized based on annotation and literature including MeSH terms, gene ontology (GO) and pathways, as described (Scherf et al., 2005; Frisch et al., 2009). Out of the 3,738 disease terms found in the MeSH database, we selected only one term relevant for our study, “trypanosomosis” (MeSH-Term ID: c03.752.300.900), using bovine as our reference species. The list of individual genes in association with bovine trypanosomosis as found in the scientific literature was filtered for significance to avoid random matches (Buza et al., 2014). Based on the parameters defined above, we selected a total of 25 genes that are significantly regulated and associated with our disease term based on the MeSH *p*-value (*p* = 1.77E-32). The 25 gene sets were then used as targets for further analysis (gene functions and references in Supplementary Table S1).

### Prediction and Extraction of Disease Associated Bovine miRNA

We searched for the identified 25 genes from our disease pathway and their locations within the bovine genome UMD 3.1 using the Ensembl BLAST/BLAT Genomic Sequence tool^2^. Complete sequence of the 25 genes were retrieved with their accession number: CD86 (XM_005201387), FcγR3A (NM_001077402), CD1A (NM_001105456), IL-6 (NM_173923), IFN-G (NM_174086), CD4 (NM_001103225), CXCL-8 (NM_173923), ICAM-1 (NM_174348), CSF2 (NM_174028), CD14 (XM_005209429), TLR-4 (NM_174198), LBP (NM_001038674), IL-10 (NM_174088), TLR-2 (NM_174197), CCL2 (NM_174006), MYD88 (NM_001014382), TNF (NM_173966), CD83(NM_001046590), CD80 (NM_001206439), IL-4 (NM_173921), IL-18 (NM_174091), LY96 (NM_001046517), ITGAM (NM_001039957), IL-12A (NM_174355), and MAPKAPK3 (NM_001034779). Thereafter, each gene was used as target to individually search the *Bos taurus* genome for possible miRNAs within their complete sequences (i.e., 3′-UTR, 5′-UTR and CDS) using miRWalk^3^; an online program which allows search for interactions between complete gene sequences and miRNAs using the TarPmiR algorithm. To avoid false positive miRNAs in our study, three other miRNA-target prediction software (TargetScan^4^, miRDB^5^ and miRBase^6^) were used to predict the miRNA and targets interaction for each gene. Only miRNAs that were confirmed in the three databases were included for further analysis. All miRNAs that were found from each gene target were partitioned into three regions; the 5′-UTR, CDS and 3′-UTR in order to examine the variation in miRNA abundance at each region of the targets. In order to identify common miRNAs among the three regions of each gene sequence, we sorted the matches using a web- based Venn diagram application^7^.

### Network Analysis, Interaction of miRNA and Target Location of the Chromosome

Using the Ensembl BLAST/BLAT and the miRBase, we locate the position of each miRNA and their targets on the chromosomes within the bovine genome. All mature miRNAs sequences of our targets were retrieved; genes located on the same chromosome were pooled together for us to identify concomitant miRNAs co-regulating one or more targets with the aid of an online Venn diagram^8^. miRNA-target network was constructed with most significant genes in the disease pathway using miRNet; an online platform that integrate miRNAs, targets and their functions^9^ (Fan and Xia, 2018).

### Analysis of 7 Innate Immune Genes Associated With Bovine Trypanosomosis

We selected seven innate immune genes among the 25 that are responding in bovine trypanosomosis pathway for further analysis. Briefly, we used the accession numbers of TLR-4 (NM_174198), ITGAM (NM_001039957), ICAM-1 (NM_174348), CD14 (XM_005209429), TLR-2 (NM_174197), LBP (NM_001038674), and TNF-α (NM_173966) to query the databases of miRWalk^10^ and miRBase^11^ in order to retrieve the mature and precursor miRNA sequences within the targets for further analysis. We selected only common miRNAs from the two databases as candidates for further analysis. Based on the number of targets by a miRNA, we selected the top 10 miRNAs regulating four to five targets. It is assumed that a miRNA with multiple targets is probably a key miRNA in the innate immune system under study (Newman and Hammond, 2010; Wang, 2010). We also compared the miRNA abundance at 5′-UTR, CDS and 3′-UTR of the selected top 10. This is also necessary assuming the regions with the most miRNAs will be more regulated and have greater impact on disease outcome.

### Evolutionary Trace and Phylogenetic Analysis of Conserved Bovine miRNA With Other Species

In order to determine the evolutionary trace and conservation of the identified bovine miRNA from the seven innate immune response genes, we selected the two miRNAs that showed the highest conservation from the 5′-UTR and 3′-UTR and 10 from the CDS being that it presented the most abundant miRNAs. Here, precursor miRNA sequences were retrieved from the miRBase database (see footnote 11); each bovine miRNA sequence was used for BLAST search with Ensembl BLAST/BLAT Genomic Sequence tool^12^. Homologous sequences from other species for each bovine miRNA were downloaded from the database for further analysis. Multiple sequence alignment (MAS) was performed using MEGA vs7 and Neighbor joining (NJ) phylogenetic tree was constructed with Cluster W to determine the nucleotide substitution and evolutionary divergence among the species. It is assumed that the most conserved miRNAs would be candidates for disease studies.

### Gene Ontology and Functional Analysis of the Most Conserved Bovine miRNAs in Innate Immune System

We performed functional enrichment analysis using gene ontology (GO) term^13^ and DIANA tools^14^ for the topmost conserved miRNAs at the 3′UTR, 5′UTR and CDS of the 7 innate immune response genes to gain understanding of the possible biological processes and physiological pathways regulated by these miRNAs according to Paraskevopoulou et al. (2013). We used 13 topmost miRNAs from the 7 innate immune genes and 6 others that are significantly conserved from CXCL-8, TLR-4, and MAPKAPK3 (Supplementary Table S2), giving a total of 19 miRNAs to search the gene ontology database to collect the relevant biological processes and the GO terms. A higher stringency was imposed by considering only the GO terms with *p* < 0.001 as significantly enriched. Where GO terms were not found for certain bovine miRNAs, closely related and annotated miRNAs in other species were used as functional homologs.

## Results

### Identification of Candidate miRNAs and Gene Targets Associated With Bovine Trypanosomosis

**FIGURE 1:**
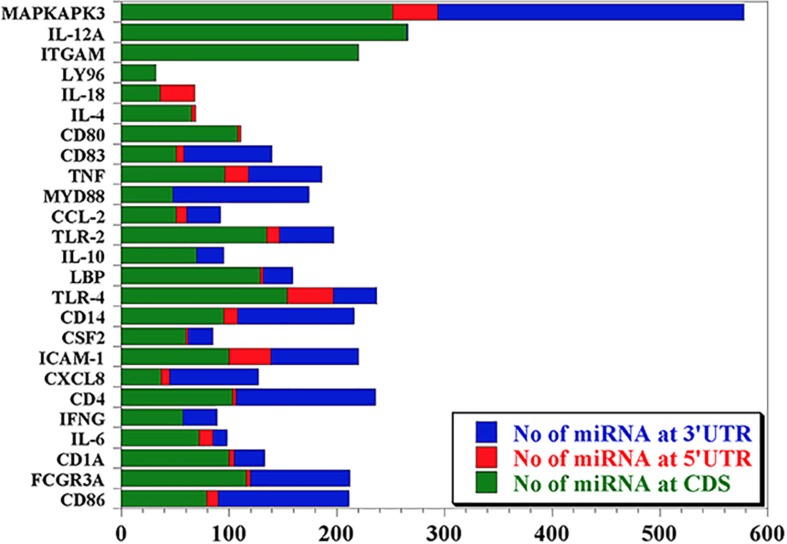
Predicted miRNA abundance targeting 25 genes significantly regulated during bovine trypanosomosis.

Our data mining revealed a total of 25 genes which were significantly regulated during bovine trypanosomosis. From the prediction analysis, we identified 4,251 bovine miRNAs that target these 25 genes spanning the 3′UTR, 5′UTR and the CDS regions (Table 1). The number of miRNAs per gene ranges from 32 as seen in LY96 to 578 in MAPKAPK3 gene. As shown in Figure 1 and Table 1, about 8 other genes showed a higher density with over 200 miRNAs. It is observed that ICAM-1 and ITGAM genes had similar miRNA number (220).

**TABLE 1:** List of significantly regulated genes during bovine trypanosomiasis and the number of miRNA at the 3′UTR, 5′UTR and CDS regions.

**S/No**	**miRNA Target**	**Accession #**	**Number of miRNA at 3′UTR**	**Number of miRNA at 5′UTR**	**Number of miRNA at CDS**	**Total miRNA**	**Chromosome number**	**Location on chromosome**
1	CD86	XM_005201387	121	11	79	211	1	66542297–66612271
2	CD80	NM_001206439	0	3	108	111	1	64301045–64324140
3	IL-12A	NM_174355	1	0	265	266	1	107432816–107440698
4	FcγR3A	NM_001077402	92	4	116	212	3	8000142–8008338
5	CD1A	NM_001105456	28	5	100	133	3	11858509–11863336
6	IL-6	NM_173923	13	13	72	98	4	31454662–31459218
7	IFN-γ	NM_174086	32	0	57	89	5	45624365–45629433
8	CD4	NM_001103225	129	4	103	236	5	103630890–103655348
9	CXCL8	NM_173923	82	8	37	127	6	88810335–88814655
10	ICAM-1	NM_174348	81	39	100	220	7	14813516–14824624
11	CSF-2	NM_174028	23	2	60	85	7	22398891–22401350
12	CD14	XM_005209429	108	13	95	216	7	51762838–51765825
13	IL-4	NM_173921	0	4	65	69	7	21696091–21704293
14	TLR-4	NM_174198	40	43	154	237	8	107057606–107069056
15	LBP	NM_001038674	27	3	129	159	13	67214225–67247921
16	LY96	NM_001046517	0	0	32	32	14	37240179–37274547
17	IL-18	NM_174091	0	32	36	68	15	22475462–22502857
18	IL-10	NM_174088	25	0	70	95	16	4550747–4555407
19	TLR-2	NM_174197	50	12	135	197	17	3953755–3967506
20	CCL-2	NM_174006	31	10	51	92	19	15902726–15905419
21	MYD88	NM_001014382	126	0	48	174	22	11609261–11613877
22	MAPKAPK3	NM_001034799	284	42	252	578	22	49711038–49740804
23	TNF-α	NM_173966	68	22	96	186	23	27716111–27719104
24	CD83	NM_001046590	82	7	51	140	23	42654473–42676909
25	ITGAM	NM_001039957	0	0	220	220	25	27343250–27382467

*Significantly regulated genes were arranged in order of chromosome number (lowest to the highest).*

**FIGURE 2:**
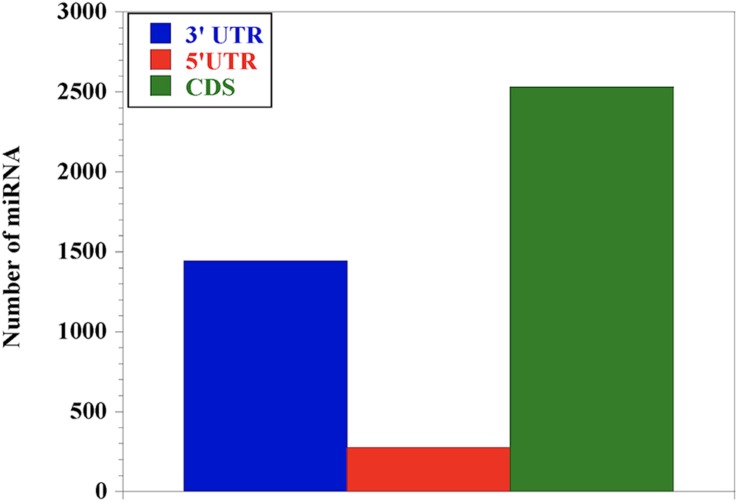
Distribution of miRNA abundance at the 3′UTR, 5′UTR and CDS of the 25 genes responding during bovine trypanosomosis. Our results show that miRNAs are relatively abundant in the coding sequence (CDS) region, indicating the importance of this region in gene regulation, followed by the 3′UTR. The 5′UTR had the least number of miRNAs.

In order to understand the distribution of the miRNAs across the nucleotide sequence of each gene, we performed miRNA-target prediction for the 3′UTR, 5′UTR and the CDS regions using miRWalk. Generally, the CDS region presents the highest number of miRNAs for most genes followed by the 3′UTR, while the 5′UTR has the lowest (Figure 2). We observed that genes like LY96 and ITGAM have no miRNA at both 3′UTR and 5′UTR; CD80, IL-4 and IL-18 have no miRNA at the 3′UTR, with IL-12A, IFN-γ, IL-1- and MYD88 presenting no miRNA at the 5′UTR.

To examine the most conserved miRNAs across the 2 or 3 regions, we compared the overlapping miRNAs for each gene, with the assumption that miRNA present at two or three regions will be conserved with important biological inferences. Notably, there are more matches of miRNAs between the 3′UTR and CDS region with FcγR3A, CD14 and MAPKAPK3 genes presenting 18, 20 and 99 matches respectively while CD80, IL-4, IL-18, LY96, ITGAM and IL-12A had no match of miRNA at the 3 regions (Table 2 and Supplementary Table S2). Overall, we observed that only CXCL-8, TLR-4, and MAPKAPK3 genes had conserved miRNAs present in their 3′UTR, 5′UTR and the CDS regions; bta-miR-2454-5p in CXCL-8, bta-miR-2328-3p in TLR-4, bta-miR-1777a, bta-miR-652 and bta-miR-7863 in MAPKAPK3 (Supplementary Table S2).

**TABLE 2:** Overlapping bovine miRNAs at the 3′UTR, 5′UTR and CDS.

**S/No**	**Gene**	**3′UTR 5′UTR match**	**3′UTR CDS match**	**5′UTR CDS match**	**3′UTR 5′UTR CDS match**
1	CD86	3	13	1	0
2	FcγR3A	1	18	0	0
3	CD1A	2	6	0	0
4	IL-6	0	2	1	0
5	IFN-γ	0	7	0	0
6	CD4	0	13	1	0
7	CXCL8	1	10	0	1
8	ICAM-1	2	12	6	0
9	CSF-2	0	2	0	0
10	CD14	6	20	0	0
11	TLR-4	4	7	13	1
12	LBP	0	5	1	0
13	IL-10	0	4	0	0
14	TLR-2	0	7	4	0
15	CCL-2	0	2	2	0
16	MYD88	0	7	0	0
17	TNF	2	11	3	0
18	CD83	0	5	1	0
19	CD80	0	0	0	0
20	IL-4	0	0	0	0
21	IL-18	0	0	5	0
22	LY96	0	0	0	0
23	ITGAM	0	0	0	0
24	IL-12A	0	0	0	0
25	MAPKAPK3	18	99	10	3

*UTR, untranslated region; CDS, coding sequence; red, indicate genes with matches and the numbers at the 3′UTR, 5′UTR and CDS regions.*

### Conserved Bovine miRNAs With Different Chromosomes

**FIGURE 3:**
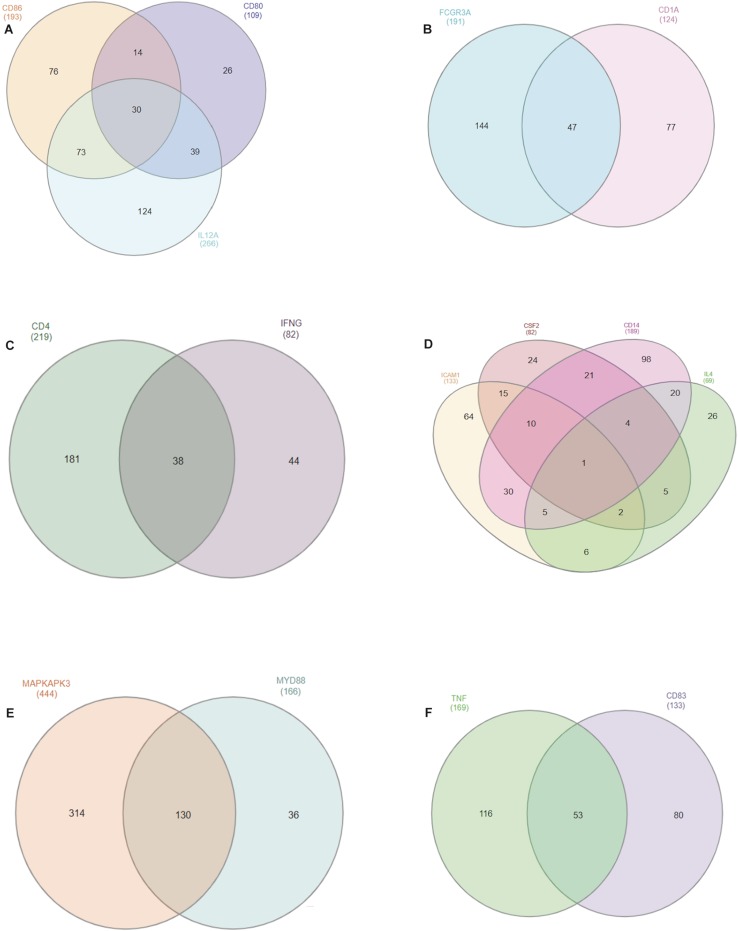
Venn diagram of conserved bovine miRNAs within different chromosomes (**A:** chromosome 1; **B:** chromosome 3; **C:** chromosome 5; **D:** chromosome 7; **E:** chromosome 22; **F:** chromosome 23). Overlapping regions in the Venn diagram represent the number of conserved miRNAs between one or two genes in the same chromosome.

We gathered miRNA from genes located on the same chromosome and we identified common miRNAs which may give biological relevance to our study. In all, we found a total of 15 out of 25 selected genes located on the same chromosome with chromosome 7 having 4 genes (CSF2, CD14, ICAM-1, and IL-4), followed by chromosome 1 possessing 3 genes (CD80, CD86, and IL-12A) while chromosomes 3, 5, 22 and 23 have 2 genes each in common (Figures 3A–F). MAPKAPK3 and MYD88 on chromosome 22 has the highest number of common miRNAs with a total of 130; while TNF-α and CD83 on chromosome 23 have 53 miRNAs in common. Thirty common miRNAs are presented between CD80, CD88, and IL-12A.

### Network Analysis of Bovine miRNA-Target Interactome During Bovine Trypanosomosis

**FIGURE 4:**
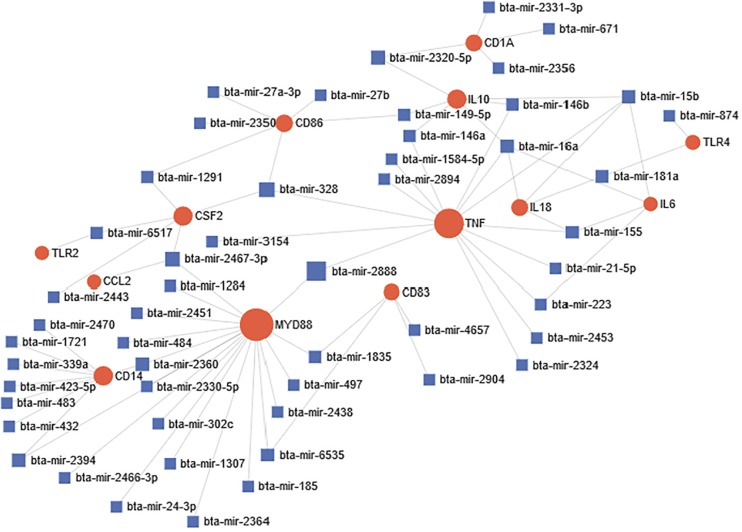
Predicted miRNA-target interactome network during bovine trypanosomosis. Circular node shape with red color represents all significantly connected genes within the network during the disease condition. Node size simulates the number of interactions, which is directly proportional. Each miRNA in the network is represented by square shape and color blue. Most important miRNA nodes joined one or two genes together thereby affecting their expression simultaneously.

miRNA-target network was constructed in order to identify significant association and connections between miRNAs and different targets during bovine trypanosomosis. Out of the 25 selected genes and 4,251 miRNAs that were subjected to network analysis, only 13 genes and 54 miRNAs were significant connected in our network (Figure 4). Overall, based on the size of each node, CD14, MYD88, TNF-α, and IL-10 were significant nodes detected from the network analysis. Likewise, bta-mir-2888, bta-mir-2394, bta-mir-1284, and bta-mir-2467-p were major miRNA nodes connecting two or more genes in the network.

### Prediction of Conserved miRNAs-Target Interaction in 7 Innate Immune Response Genes

We focused on identifying miRNAs that are concomitantly present in the 7 innate immune response genes among the list of 25 genes that are significantly responding to bovine trypanosomosis. Here, we postulate that bovine miRNAs targeting the greatest number of genes will be potential candidates as biomarkers for disease diagnosis or treatment. From Tables 3–5, we present the prediction analysis of miRNA-target interactions and their matured sequences. We showed the top 10 bovine miRNAs and their targets based on the 3′UTR, 5′UTR, and the CDS regions. Our selection is based on the miRNAs that target between 3-5 genes out of the selected 6 innate immune response genes. We observe that the CDS region has miRNAs that target the most genes; 5 out of the 7 genes at a time. For example, bta-miR-2349 targets CD14, ITGAM, TLR-2, TLR-4, and TNF-α while bta-miR-193a-5p targets ICAM-1, ITGAM, LBP, TLR-2, and TNF-α (Table 3). Following the CDS region is the 3′UTR, while the 5′UTR has the least number of miRNAs, targeting less than 4 genes (Tables 4, 5). bta-miR-2460 from the 3′UTR is demonstrated to target CD14, ICAM-1, TLR-4, and TNF-α genes, while bta-miR-2374 are targeting CD14, ICAM-1, LBP, and TLR-4 gene sets (Table 4), indicating they are the topmost in this region. Only bta-miR-2392 is targeting ICAM-1, TLR-2, TLR-4 and TNF-α from the 5′UTR (Table 5).

**TABLE 3:** Top 10 MicroRNA at the CDS region and their target genes.

**S/No**	**MicroRNA**	**Mature accession**	**Mature sequence**	**Size**	**Combined target genes**
1	bta-miR-2349	MIMAT0011884	UGGCACUUCUGGUCUCAGACUCA	38–60	CD14, ITGAM, TLR-2, TLR-4, TNF
2	bta-miR-3602	MIMAT0016937	GUGUUGGGAUCACCGCGGUAA	18–38	CD14, ITGAM, LBP, TLR-2, TLR-4
3	bta-miR-449d	MIMAT0011962	GAAGGCUGUGUGCUGUGGAG	16–35	CD14, ITGAM, LBP, TLR-2, TLR-4
4	bta-miR-2316	MIMAT0011837	ACUCCGGCCUGGACUGCGGCGGG	10–32	CD14, ICAM-1, TLR-2, TLR-4, TNF
5	bta-miR-4657	MIMAT0036970	AAUGUGGAAGUGGUCUGAGGCAU	1–23	CD14, ICAM-1, LBP, TLR-2, TNF
6	bta-miR-2888	MIMAT0013846	GGUGGGGUGGGGGGGUUGG	47–65	ITGAM, LBP, TLR-2, TLR-4, TNF
7	bta-miR-2295	MIMAT0011803	UCGGGGUGGGAGGAAGGUUCU	43–63	ITGAM, LBP, TLR-2, TLR-4, TNF
8	bta-miR-193a-5p	MIMAT0003795	AACUGGCCUACAAAGUCCCAGU	49–70	ICAM-1, ITGAM, LBP, TLR-2, TNF
9	bta-miR-2422	MIMAT0011989	UUGAGGGGACUGAGGUGCGGAG	4–25	ICAM-1, ITGAM, LBP, TLR-2, TNF
10	bta-miR-2456	MIMAT0012041	ACGCACUGUCCUGGGAAGUGG	45–65	ICAM-1, ITGAM, LBP, TLR-4, TNF

**TABLE 4:** Top 10 MicroRNA at the 3′ untranslated region and their target genes.

**S/No**	**MicroRNA**	**Mature accession**	**Mature sequence**	**Size**	**Combined target genes**
1	bta-miR-2460	MIMAT0012045	UGGAGCUCUUGAGGCCUGGCAU	6–27	CD14, ICAM-1, TLR-4, TNF
2	bta-miR-2374	MIMAT0011920	UUGGGGCUGGGGAGAGGCGGG	45–65	CD14, ICAM-1, LBP, TLR-4
3	bta-miR-3431	MIMAT0017394	CCUCAGUCAGCCUUGUGGAUGU	21–42	CD14, ICAM-1, TLR-2
4	bta-miR-2343	MIMAT0011878	AAGGGGAGACGGUGGAACUUAU	11–32	CD14, ICAM-1, TLR-4
5	bta-miR-2308	MIMAT0011820	UUGGGCUUGCAGCAGAGAGUAA	44–65	CD14, ICAM-1, TLR-4
6	bta-miR-2328-3p	MIMAT0011857	GCCCCCUCCCUUGGUCGCCGG	10–30	CD14, TLR-4, TNF
7	bta-miR-328	MIMAT0009287	CUGGCCCUCUCUGCCCUUCCGU	61–82	CD14, TLR-4, TNF
8	bta-miR-2888	MIMAT0013846	GGUGGGGUGGGGGGGUUGG	47–65	CD14, LBP, TLR-4
9	bta-miR-3141	MIMAT0024573	GAGGGCGGGUGGAGGAGG	77–94	CD14, ICAM-1, LBP
10	bta-miR-320b	MIMAT0011991	AGCUGGGUUGAGAGGGUGGU	43–62	CD14, LBP, TNF

**TABLE 5:** Top 10 MicroRNA at the 5′ untranslated region and their target genes.

**S/No**	**MicroRNA**	**Mature accession**	**Mature sequence**	**Size**	**Combined target genes**
1	bta-miR-2392	MIMAT0011945	AUGGAUGGGGGUGAGGGGUGCA	45–66	ICAM-1, TLR-2, TLR-4, TNF
2	bta-miR-181b	MIMAT0003793	AACAUUCAUUGCUGUCGGUGGGUU	36–59	CD14, TLR-2
3	bta-miR-1249	MIMAT0009976	ACGCCCUUCCCCCCCUUCUUCA	41–62	CD14, ICAM-1
4	bta-miR-6120-5p	MIMAT0024590	CUGUUCCCGUUUUUCACAUGUG	3–24	CD14, TNF
5	bta-miR-329b	MIMAT0009289	AGAGGUUUUCUGGGUUUCUGUUU	13–35	CD14, TNF
6	bta-miR-27a-5p	MIMAT0012532	AGGGCUUAGCUGCUUGUGAGCA	14–35	TLR-2, TNF
7	bta-miR-345-3p	MIMAT0012535	CCUGAACUAGGGGUCUGGAG	55–74	ICAM-1, TLR-4
8	bta-miR-7865	MIMAT0030450	CAGGGAGGGCAGGGGAGGG	1–19	TLR-4, TNF
9	bta-miR-6526	MIMAT0025556	UCCUGUGCCUCGAAUGGGUAUG	48–69	TLR-4, TNF
10	bta-miR-2443	MIMAT0012018	UGAGGGCAGGACCGUAUGAGGUGU	9–32	LBP, TLR-4

Table 6 shows the number of miRNAs common to a gene pair from each region. Our analyses reveal that the highest number of bovine miRNAs common between two genes are located at the CDS region. There are 55 miRNAs shared between TLR-4 and ITGAM, 54 between LBP and ITGAM, 52 between TLR-2 and ITGAM, 44 between ITGAM and ICAM-1, and 40 between TNF-α and ITGAM. Notably, CD14 and TNF-α have the highest common miRNA (15) at the 3′UTR followed by 10 between CD14 and TLR-4 (Table 6).

**TABLE 6:** Number of concomitant miRNAs at the 3′UTR, 5′UTR and CDS regions of seven innate immune response genes.

**Gene target**		**CD14**	**TLR-2**	**TLR-4**	**ITGAM**	**ICAM-1**	**TNF-α**	**LBP**
CD14	3′ UTR	71	6	10	0	12	15	5
	5′ UTR	9	1	0	0	1	2	0
	CDS	27	23	26	34	17	23	17
TLR-2	3′ UTR		27	1	0	9	8	1
	5′ UTR		9	1	0	1	2	0
	CDS		31	33	52	26	29	36
TLR-4	3′ UTR			20	0	7	6	6
	5′ UTR			37	0	2	3	2
	CDS			42	55	24	35	33
ITGAM	3′ UTR				0	0	0	0
	5′ UTR				0	0	0	0
	CDS				57	44	40	54
ICAM-1	3′ UTR					48	7	7
	5′ UTR					34	3	0
	CDS					15	22	27
TNF-α	3′ UTR						37	4
	5′ UTR						14	0
	CDS						13	32
LBP	3′ UTR							10
	5′ UTR							1
	CDS							28

### Study of Evolutionarily Conserved Bovine miRNA Among the 7 Innate Immune Response Genes and Other Species

**FIGURE 5:**
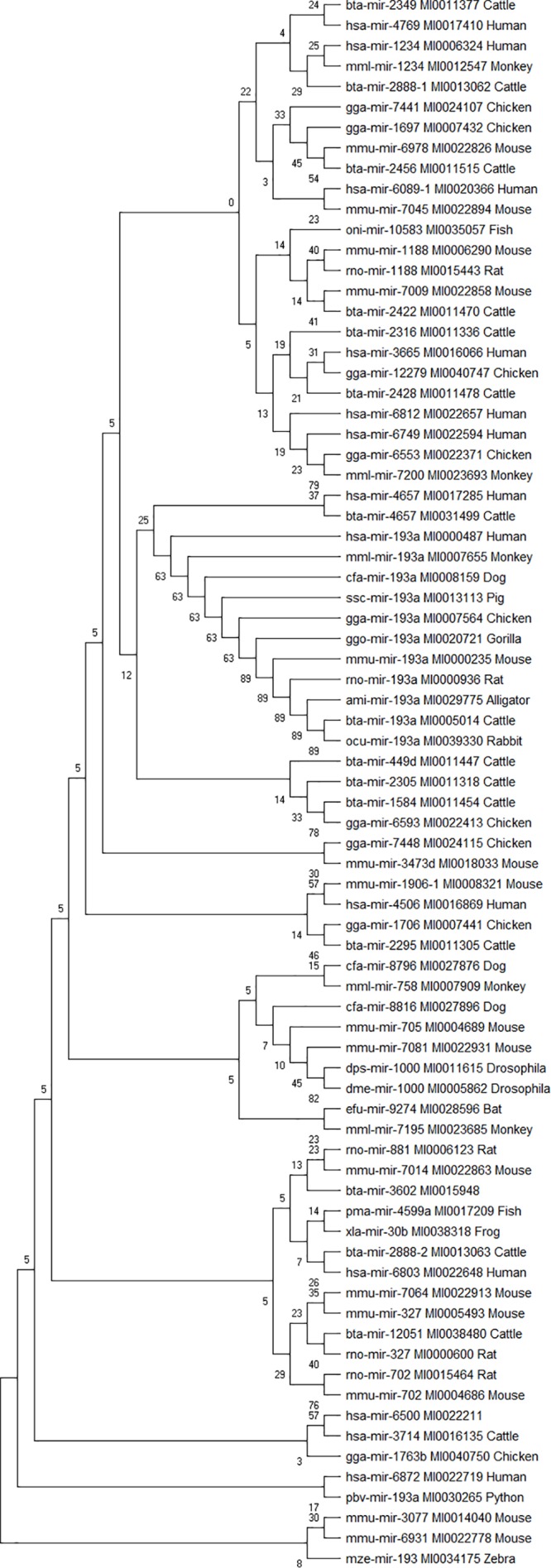
Phylogenetic tree of 13 bovine miRNA and their homologs in other species. The evolutionary trace was inferred using the Neighbor-Joining method. The tree is drawn to scale, with branch lengths in the same units as those of the evolutionary distances used to infer the phylogenetic tree. The evolutionary distances were computed using the p-distance method and are in the units of the number of nucleotide sequence differences per site. The analysis involved 77 nucleotide sequences.

**FIGURE 6:**
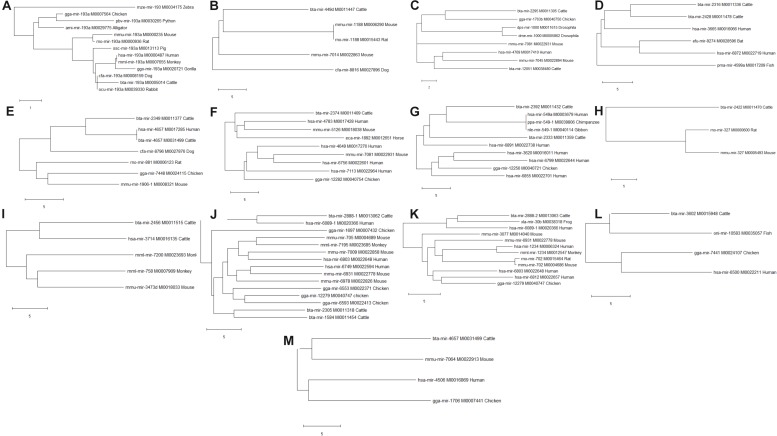
Evolutionarily trace of conserved bovine miRNA among the 7 innate immune genes and other species (**A:** bta-mir-193a; **B:** bta-mir-449d; **C:** bta-mir-2295; **D:** bta-mir-2316; **E:** bta-mir-2349; **F:** bta-mir-2374; **G:** bta-mir-2392; **H:** bta-mir-2422; **I:** bta-mir-2456; **J:** bta-mir-2888-1; **K:** bta-mir-2888-2; **L:** bta-mir-3602; **M:** bta-mir-4657).

Phylogenetic analyses were performed to examine the evolutionarily conserved bovine miRNAs with other species. To be more stringent, only miRNAs that target 4 out of 7 innate immune response genes were included in the analysis (one from the 5′UTR, 2 from 3′UTR and 10 from the CDS). Analysis of all the 10 miRNAs at the CDS with their homologous sequences from other species identified bta-mir-193a as highly conserved with human, monkey, dog, pig, chicken, gorilla, mouse, rat, rabbit, and alligator (Figure 5). In the same manner, bovine bta-mir-4657 clusters with a human homolog hsa-mir-4657, bta-mir-2888-2 with hsa-mir-6803 and bta-mir-2349 with hsa-mir-4769 (Figure 5). Furthermore, we individually searched for the conserved homologs of the 13 miRNAs selected for stringency (one from the 5′UTR, 2 from 3′UTR, and 10 from the CDS) in order to determine their evolutionary trace with other species. Figures 6A–M show different clusters of each miRNA with their homologs from other species. Based on the number of homologs found with other species, bta-mir-2888-1 (Figure 6J) with 14 homologs has the highest followed by bta-mir-193a with 13 (Figure 6A), bta-mir-2888-2, bta-mir-2392, and bta-mir-2349 have 11, 9 and 8 homologs respectively (Figures 6E,G,K). bta-mir-2422 presented the least homolog with rat (rno-mir-327) and mouse (mmu-mir-327) (Figure 6H).

### Enrichment Analysis and GO Terms of the Conserved miRNAs

In order to better elucidate the possible biological and physiological processes regulated by the identified bovine miRNAs, 19 predicted conserved miRNAs were subjected to GO term analysis using the sub-term biological process as the focus. Topmost GO terms were selected for each tested miRNA as significantly enriched based on the *p*-value of less than 0.001. From the analysis, we found only 10 out of the 19 bovine miRNAs being functionally annotated with GO terms (Table 7). We infer the functional annotation of the remaining 9 bovine miRNAs from closely related species (in parentheses) based on the homology between 95-100%. We found that bta-mir-2460, bta-mir-193a, bta-mir-2328-3p, bta-mir-652, bta-mir-2392, and bta-mir-2454 are significantly involved with gene silencing (GO:0035195) and RNA-induced silencing complex (GO:0016442); bta-mir-2295 is involved with signal transduction in response to DNA damage (GO:0042770); bta-mir-2422 is involved with regulation of humoral immune response mediated by circulating immunoglobulin (GO:0002923), and bta-mir-2888-2 is involved with 2-aminobenzenesulfonate metabolic process (GO:0018868), among others (Table 7).

**TABLE 7:** Gene Ontology of the top conserved predicted miRNA-target interaction.

**miRNA**	**Regional location**	**GO term (biological process)**	**GO ID**	***P*-value**	**Target genes**
bta-mir-2374	3′UTR (human)	Cellular nitrogen compound metabolic process	GO:0034641	5.817e-07	CD14, ICAM-1, LBP, TLR-4
bta-mir-2460	3′UTR	Gene silencing by miRNA	GO:0035195	2.638e-03	CD14, ICAM-1, TLR-4, TNF
bta-mir-2392	5′UTR	RNA-induced silencing complex	GO:0035068	8.283e-05	ICAM-1, TLR-2, TLR-4, TNF
bta-mir-193a	CDS	Gene silencing by miRNA	GO:0035195	0.0076	CD14, ITGAM, TLR-2, TLR-4, TNF
bta-mir-449d	CDS (mouse)	Cellular differentiation	GO:0030154	0.0014	CD14, ITGAM, LBP, TLR-2, TLR-4
bta-mir-2295	CDS (chicken)	Signal transduction in response to DNA damage	GO:0042770	0.0018	CD14, ITGAM, LBP, TLR-2, TLR-4
bta-mir-2316	CDS (human)	Regulation of translation in response to stress	GO:0043555	0.0168	CD14, ICAM-1, TLR-2, TLR-4, TNF
bta-mir-2349	CDS (human)	Cellular protein modification process	GO:0006464	0.0373	CD14, ICAM-1, LBP, TLR-2, TNF
bta-mir-2422	CDS (human)	Regulation of humoral immune response mediated by circulating immunoglobulin	GO:0002923	0.0479	ITGAM, LBP, TLR-2, TLR-4, TNF
bta-mir-2456	CDS (mouse)	Cellular protein modification process	GO:0006464	7.549e-07	ITGAM, LBP, TLR-2, TLR-4, TNF
bta-mir-2888-1	CDS	Dihydrosphingosine-1-P pathway	GO:0006648	0.0069	ICAM-1, ITGAM, LBP, TLR-2, TNF
bta-mir-2888-2	CDS	2-aminobenzenesulfonate metabolic process	GO:0018868	2.720e-03	ICAM-1, ITGAM, LBP, TLR-2, TNF
bta-mir-3602	CDS (human)	Transcription regulator activity	GO:0010467	3.066e-05	ICAM-1, ITGAM, LBP, TLR-4, TNF
bta-mir-4657	CDS (human)	Cellular protein modification process	GO:0006464	0.0028	CD14, ITGAM, TLR-2, TLR-4, TNF
bta-mir-2454	3′UTR 5′UTR CDS	RNA-induced silencing complex	GO:0016442	7.857e-05	CXCL-8
bta-mir-2328-3p	3′UTR 5′UTR CDS	Gene silencing by miRNA	GO:0000115	0.0062	TLR-4
bta-mir-1777a	3′UTR 5′UTR CDS	Micro-ribonucleoprotein complex	GO:0016442	0.0012	MAPKAPK3
bta-mir-652	3′UTR 5′UTR CDS	Gene silencing by miRNA	GO:0035195	0.0016	MAPKAPK3
bta-mir-7863	3′UTR 5′UTR CDS	Negative regulation of chronic inflammatory response	GO:0008283	0.0887	MAPKAPK3

## Discussion

Identification of molecular biomarkers associated with diseases is important in early disease diagnosis, drug target and treatment. miRNAs are increasingly emerging as molecular biomarkers because of their essential role in gene regulation both in animals and plants during disease condition (Buza et al., 2014; Agarwal et al., 2015; Scheel et al., 2017). In this study, we identified and elucidated conserved miRNAs from a list of gene targets important in innate immune response during bovine trypanosomosis through computational prediction analysis. Previous studies have focused on the 3′UTR region to study the miRNA-target interactions while few others suggested that miRNAs can anneal within the 3′UTR, 5′UTR or CDS regions of their target to regulate gene expression (Erhard et al., 2014; Yang et al., 2017; da Silveira et al., 2018). This study however brings a unique perspective through identification of conserved miRNAs targeting immune genes responding during bovine trypanosomosis by analyzing the complete sequence of the gene which include the 3′-, 5′ UTRs and the CDS. Our study showed a significantly higher density of miRNA binding sites at the CDS region for all genes, identifying it as an important region which may play a significant regulatory role in expression during the disease condition. Studies have shown that target genes having many miRNA binding sites at the CDS region can be easily degraded (Schnall-Levin et al., 2011; Hausser and Zavolan, 2014). Likewise, Ott et al. (2011) and Chi et al. (2009) discovered that there are increasing miRNA target sites at the CDS region of mammalian transcripts during immunoprecipitation studies. This observation is in line with our results, suggesting that CDS sites may have specific function in regulating gene expression rather than the previous focus on the 3′UTR (Hausser et al., 2013). Brummer and Hausser (2014) stated that the CDS sites may enhance the repertoire of miRNA regulation than the 3′UTR sites because of their interaction with alternative polyadenylation, alternative splicing and the general gene architecture.

In addition, our study identified significant overlaps of miRNA binding sites between the 3′UTR and CDS regions, indicating possible evolutionary conservation of these regulatory elements between the two regions (Hurst, 2006; Gaidatzis et al., 2007; Forman et al., 2008). Published reports have shown that miRNAs simultaneously targeting both 3′UTR and CDS regions of mRNAs are more regulated than those targeted by 3′UTR only (Fang and Rajewsky, 2011; Bazzini et al., 2012). Therefore, we adjudge that conserved miRNAs found targeting both regions have undergone selective pressure, and are therefore evolutionarily conserved, which may induce mRNA translational inhibition and degradation of immune gene response during bovine trypanosomosis.

Furthermore, we found significant miRNAs cluster for genes located on the same chromosome, the most indication suggesting evolutionary conservation and functional roles on their targets. Thus, there is the possibility that expression of two or more genes on the same chromosome can be co-regulated by a single miRNA, and this observation might be important for quantifying gene regulation during disease condition or as targets for treatment. Pande et al. (2018) and Hanif et al. (2018) support the finding that miRNAs clusters across cattle genome have variable functions through large evolutionary distance.

Additionally, our study showed networks between miRNAs and target genes, which are significantly connected during bovine trypanosomosis. From our network interactome, CD14, TLR-2, TLR-4, IL10 MYD88, TNF-α, bta-mir-2360, bta-mir-1835, bta-mir-2888, bta-mir-146b and bta-mir-2320-5p amongst others are important drivers and connectors within the network, indicating they are major players during disease condition. Studies have shown that molecular interactions between genes, proteins, RNA and other biological molecules contribute to cellular performance and homeostasis (Govindaraju et al., 2012; Ji et al., 2018; Wang et al., 2018). The co-expression of these miRNAs within the network may regulate the interaction of certain gene sets such as MYD88 and TNF-α, CD14 and TLR-2, IL-18, and TLR-4, among others. Studies to date have demonstrated the importance of CD14 and TLR gene family in facilitating pattern recognition of pathogen-associated molecules and to elicit innate immune response against viruses, bacteria, fungi, and protozoa (Seabury et al., 2010; Ojurongbe et al., 2019; Morenikeji and Thomas, 2019b). Likewise, MYD88 is a signal transduction adaptor, transferring signals from interleukin-1 (IL-1) receptors and Toll-like receptors, and driving macrophage release of cytokines, imperative in innate immune response to exogenous pathogenic stimuli. Therefore, a significant overlap of miRNAs targeting these genes altogether could work synergistically as putative markers for disease discovery, characterization and therapeutic manipulations for bovine trypanosomiasis treatment.

Moreover, among the 7 innate immune response genes considered in this study, our functional analysis show that bta-mir-2460, bta-mir-193a, bta-mir-2349, bta-mir-3602, bta-mir-2454, and bta-mir-652 are significantly enriched within biological processes such as gene silencing, RNA-induced silencing complex, and cellular protein modification process. These miRNAs can potentially regulate CD14, ITGAM, LBP, TLR-2, TLR-4, and TNF-α genes and their expression might be key regulatory factors that determine disease susceptibility, tolerance or resistance in cattle. We have shown that CD14 is particularly implicated in disease tolerance among cattle with bovine trypanosomosis, with significant expression among trypanotolerant animals and the reverse among trypanosusceptible ones (Morenikeji and Thomas, 2019a). Notably, some studies have also shown that bta-mir-2460, bta-mir-193a, and bta-mir-652 are significantly involved in biological regulation, cellular and metabolic process, cell death, establishment of localization, and growth, thereby supporting their significant role in disease development (Jin et al., 2013; Schanzenbach et al., 2017; Li et al., 2018).

The significant number of targets by each miRNA depicts evidence of their evolutionary conservation with the implication that they play an essential role in gene regulation during bovine trypanosomosis. Yang et al. (2012) and Zheng et al. (2014) have reported the significant role of miRNAs in fine-tuning innate immune gene expression in Holstein cows during heat stress. Remarkably, bta-mir-2888-1 and bta-mir-2888-2 together targets ICAM-1, ITGAM, LBP, TLR-2, and TNF-α to respond to dihydrosphingosine-1-P pathway and 2-aminobenzenesulfonate metabolic process, which are important regulators of physiological biosynthesis, cell survival, proliferation, and cell-cell interactions (Bu et al., 2008). Previous studies have shown that ICAM-1, TLR-4 and ITGAM were implicated in early inflammation and signaling in critical pathways regulating bovine innate immunity (Boulougouris et al., 2019). As seen from our study therefore, bta-mir-2888-1 and bta-mir-2888 would be significant biomarkers that regulate the expression of these genes during disease condition and could serve as potential therapeutic targets for bovine trypanosomosis.

Our phylogenetic analysis revealed bta-mir-193a to be highly conserved across different species ranging from human to gorilla, monkey, mouse, rat, rabbit, chicken, pig, dog, python, and zebra. This shows that bta-mir-193a has a long history of evolution among others and would be a molecular marker of choice in bovine disease studies, and as such require additional *in vitro* validation studies. Reports from other studies have shown that bta-miR-193a regulates the expression of many proinflammatory cytokines, promote apoptosis and inhibition of bacteria in mice, goat and cattle (Dilda et al., 2011; Rosenberger et al., 2012; Lawless et al., 2013; Li et al., 2018). The fact that our results show bta-mir-193a targeting CD14, ITGAM, TLR-2, TLR-4, and TNF-α strongly supports its roles in transcriptional activation of proinflammatory response and innate immune gene expression during bovine trypanosomosis. This provides compelling evidence that this miRNA could serve as a significant biomarker in disease diagnosis and treatment. Many *in vitro* studies have elucidated the significant roles of miRNAs in regulating bovine immune responses during disease conditions (Dilda et al., 2011; Lawless et al., 2013; Wang et al., 2017; Ammah et al., 2018). The fact that TLR-2 and TLR-4 are part of bta-miR-193a target suggests its involvement in Toll-like receptor signaling during host response to trypanosomosis infection.

## Conclusion

Our study provides computational evidence that certain miRNAs are conserved within the bovine genome and may be associated with regulation of immune response during bovine trypanosomosis. We propose that miRNAs targeting more genes will play greater roles in immune regulation and as such exert significant impact on disease phenotype. We also show that the CDS region has abundant miRNA binding site for all genes in this study demonstrating the importance of this region and requiring further elucidation in bovine disease studies. Additionally, we demonstrate that miRNA that co-target 3′UTR and CDS are important and capable of impacting target gene expressions in a complementary manner. Finally, our study identified some microRNAs including bta-mir-193a, bta-mir-2460, bta-mir-2349, and bta-mir-2888 amongst others, which might be important regulatory markers for diagnosis of bovine trypanosomosis as well as treatment and drug targets.

## Data Availability

All datasets generated for this study are included in the manuscript and/or the Supplementary Files.

## Author Contributions

OM and MH performed the experiment. OM, MH, AH, and BT analyzed the data. OM, AH, and BT wrote the manuscript. All authors reviewed and approved the final manuscript.

## Conflict of Interest Statement

The authors declare that the research was conducted in the absence of any commercial or financial relationships that could be construed as a potential conflict of interest.

## Footnotes

1 https://www.genomatix.de/solutions/genomatix-software-suite.html

2 http://www.ensembl.org/Tools/Blast/GenomicSeq

3 http://mirwalk.umm.uni-heidelberg.de

4 http://www.targetscan.org/vert_72/

5 http://mirdb.org/

6 http://www.mirbase.org/index.shtml

7 http://bioinfogp.cnb.csic.es/tools/venny/

8 http://bioinformatics.psb.ugent.be/software/details/Venn-Diagrams

9 https://www.mirnet.ca/miRNet/faces/upload/GeneUploadView.xhtml

10 http://mirwalk.umm.uni-heidelberg.de/search_genes/

11 http://www.mirbase.org/index.shtml

12 http://www.ensembl.org/Tools/Blast/GenomicSeq

13 http://geneontology.org/

14 http://diana.imis.athena-innovation.gr/DianaTools/index.php
