# Supplementary material for: Computational Network Analysis Identifies Evolutionarily Conserved miRNA Gene Interactions Potentially Regulating Immune Response in Bovine Trypanosomosis
Source: Front Microbiol. 2019 Aug 28;10:2010. doi: 10.3389/fmicb.2019.02010 (PMC6722470; doi:10.3389/fmicb.2019.02010)
Supplement: Supplementary file 1 [file Table_1.docx]

**Supplementary Table 1: List of immune genes significantly responding to bovine trypanosomosis and their functions**

| **S/N** | **miRNA**  **Target** | **Accession #** | **Chr #** | **Location on chromosome** | | **Gene function** | **Reference** |
| --- | --- | --- | --- | --- | --- | --- | --- |
|  |  |  |  | **Start** | **End** |  |  |
| 1 | CD86 | XM_005201387 | 1 | 66542297 | 66612271 | Production of interleukin-2 and T-lymphocyte proliferation | https://www.uniprot.org/uniprot/Q1JPC5 |
| 2 | CD80 | NM_001206439 | 1 | 64301045 | 64324140 | Cytokine production and T-cell proliferation | https://www.uniprot.org/uniprot/O46405 |
| 3 | IL-12A | NM_174355 | 1 | 107432816 | 107440698 | Stimulation of IFN-gamma production, serves as growth factor for activated T and NK cells | https://www.uniprot.org/uniprot/P54349 |
| 4 | FcγR3A | NM_001077402 | 3 | 8000142 | 8008338 | Mediation of antibody-dependent cellular cytotoxicity (ADCC). | https://www.uniprot.org/uniprot/Q2KI63 |
| 5 | CD1A | NM_001105456 | 3 | 11858509 | 11863336 | Antigen-presenting protein to T-cell receptors on natural killer T-cells. | https://www.uniprot.org/uniprot/A6QQB8 |
| 6 | IL-6 | NM_173923 | 4 | 31454662 | 31459218 | It has several of biological functions and plays an essential role in B-cells differentiation. | https://www.uniprot.org/uniprot/P26892 |
| 7 | IFN-γ | NM_174086 | 5 | 45624365 | 45629433 | It has important immunoregulatory functions and antiviral activity. it can potentiate the antiviral and antitumor effects of the type I interferons. | https://www.uniprot.org/uniprot/P07353 |
| 8 | CD4 | NM_001103225 | 5 | 103630890 | 103655348 | It plays an essential role in the immune response and serves multiple functions in responses against both external and internal offenses. | https://www.uniprot.org/uniprot/F1MJK4 |
| 9 | CXCL8 | NM_173923 | 6 | 88810335 | 88814655 | IL-8 is a chemotactic factor that attracts neutrophils, basophils, and T-cells. It is also involved in neutrophil activation. | https://www.uniprot.org/uniprot/P79255 |
| 10 | ICAM-1 | NM_174348 | 7 | 14813516 | 14824624 | ICAM proteins are ligands for the leukocyte adhesion protein LFA-1 (integrin alpha-L/beta-2). | https://www.uniprot.org/uniprot/Q95132 |
| 11 | CSF-2 | NM_174028 | 7 | 22398891 | 22401350 | It stimulates the growth and differentiation of hematopoietic precursor cells from various lineages, including granulocytes, macrophages, eosinophils and erythrocytes. | https://www.uniprot.org/uniprot/P11052 |
| 12 | CD14 | XM_005209429 | 7 | 51762838 | 51765825 | It is a coreceptor for bacterial lipopolysaccharide and mediates innate immune response to bacterial lipopolysaccharide (LPS). | https://www.uniprot.org/uniprot/Q95122 |
| 13 | IL-4 | NM_173921 | 7 | 21696091 | 21704293 | B-cell activation processes. | https://www.uniprot.org/uniprot/P30367 |
| 14 | TLR-4 | NM_174198 | 8 | 107057606 | 107069056 | It works together with LY96 and CD14 to mediate the innate immune response to bacterial lipopolysaccharide (LPS). | https://www.uniprot.org/uniprot/Q9GL65 |
| 15 | LBP | NM_001038674 | 13 | 67214225 | 67247921 | It is important in innate immune response to Gram-negative bacteria. | https://www.uniprot.org/uniprot/Q2TBI0 |
| 16 | LY96 | NM_001046517 | 14 | 37240179 | 37274547 | It binds bacterial lipopolysaccharide (LPS) from Gram-positive and Gram-negative bacteria | https://www.uniprot.org/uniprot/P58754 |
| 17 | IL-18 | NM_174091 | 15 | 22475462 | 22502857 | It is a proinflammatory cytokine that polarizes T-helper 1 (Th1) cell and natural killer (NK) cell immune responses. | https://www.uniprot.org/uniprot/Q9TU73 |
| 18 | IL-10 | NM_174088 | 16 | 4550747 | 4555407 | Major immune regulatory cytokine that acts on many cells of the immune system. | https://www.uniprot.org/uniprot/P43480 |
| 19 | TLR-2 | NM_174197 | 17 | 3953755 | 3967506 | It mediates innate immune response to bacterial lipoproteins and other microbial cell wall. | https://www.uniprot.org/uniprot/Q95LA9 |
| 20 | CCL-2 | NM_174006 | 19 | 15902726 | 15905419 | Acts as a ligand for C-C chemokine receptor. | https://www.uniprot.org/uniprot/P28291 |
| 21 | MYD88 | NM_001014382 | 22 | 11609261 | 11613877 | It is an adapter protein involved in the Toll-like receptor and IL-1 receptor signaling pathway in the innate immune response | https://www.uniprot.org/uniprot/Q599T9 |
| 22 | MAPKAPK3 | NM_001034799 | 22 | 49711038 | 49740804 | It is involved in cytokines production, cell migration, endocytosis, transcriptional regulation and chromatin remodeling. | https://www.uniprot.org/uniprot/Q3SYZ2 |
| 23 | TNF-α | NM_173966 | 23 | 27716111 | 27719104 | It stimulates cell proliferation, cell differentiation and can induce cell death of certain tumor cell lines. | https://www.uniprot.org/uniprot/Q06599 |
| 24 | CD83 | NM_001046590 | 23 | 42654473 | 42676909 | It plays an important role in antigen presentation and the cellular interactions after lymphocyte activation. | https://www.uniprot.org/uniprot/Q2KHW2 |
| 25 | ITGAM | NM_001039957 | 25 | 27343250 | 27382467 | It is a receptor for ICAM1, ICAM2, ICAM3 and ICAM4 which helps ligands for leukocyte adhesion | https://www.uniprot.org/uniprot/P32592 |
