# Supplementary material for: Computational Network Analysis Identifies Evolutionarily Conserved miRNA Gene Interactions Potentially Regulating Immune Response in Bovine Trypanosomosis
Source: Front Microbiol. 2019 Aug 28;10:2010. doi: 10.3389/fmicb.2019.02010 (PMC6722470; doi:10.3389/fmicb.2019.02010)
Supplement: Supplementary file 2 [file Table_2.docx]

**Supplementary Table 2: List of miRNAs matches at the 3’UTR, 5’UTR and CDS**

| Gene | 3’UTR 5’UTR match | 3’UTR CDS match | 5’UTR CDS match | 3’UTR 5’UTR CDS match |
| --- | --- | --- | --- | --- |
| CD86 | bta-miR-1306, bta-miR-2364, bta-miR-30b-3p | bta-miR-182, bta-miR-183, bta-miR-2284q, bta-miR-2291  bta-miR-2368-5p, bta-miR-2385-5p, bta-miR-2418, bta-miR-2425-5p, bta-miR-6528, bta-miR-6535, bta-miR-708, bta-miR-7857, bta-miR-7865 | bta-miR-2897 |  |
| FcγR3A | bta-miR-2450a | bta-miR-1225-3p, bta-miR-149-3p, bta-miR-1777b, bta-miR-1842, bta-miR-205, bta-miR-2295, bta-miR-2304, bta-miR-2313-3p, bta-miR-2313-5p, bta-miR-2326, bta-miR-2376, bta-miR-2385-5p, bta-miR-2392, bta-miR-2430, bta-miR-497, bta-miR-584, bta-miR-6529b, bta-miR-767 |  |  |
| CD1A |  | bta-miR-2343, bta-miR-2374, bta-miR-2439-5p, bta-miR-2449, bta-miR-423-5p, bta-miR-744 | bta-miR-1777a, bta-miR-2306 |  |
| IL-6 |  | bta-miR-2338, bta-miR-2392 | bta-miR-2343 |  |
| IFNγ |  | bta-mir-193a-5p, bta-mir-22-3p, bta-mir-2422, bta-mir-2471-5p, bta-mir-541, bta-mir-6534, bta-mir-93 |  |  |
| CD4 |  | bta-miR-22-3p, bta-miR-2290, bta-miR-2295, bta-miR-2324, bta-miR-2349, bta-miR-2379, bta-miR-2394, bta-miR-2403, bta-miR-2433, bta-miR-24-3p, bta-miR-412, bta-miR-7857, bta-miR-92b | bta-miR-331-3p |  |
| CXCL-8 | bta-miR-2360 | bta-let-7e, bta-miR-149-5p, bta-miR-1777b, bta-miR-2316, bta-miR-2382-5p, bta-miR-2881, bta-miR-2885, bta-miR-2899, bta-miR-2904, bta-miR-7865 |  | bta-miR-2454-5p |
| ICAM-1 | bta-miR-6520, bta-miR-665 | bta-miR-132, bta-miR-154b, bta-miR-224, bta-miR-2410, bta-miR-2422, bta-miR-2439-5p, bta-miR-2440, bta-miR-2441, bta-miR-2461-3p, bta-miR-28, bta-miR-2901, bta-miR-423-3p | bta-let-7b, bta-miR-1343-3p, bta-miR-1343-5p, bta-miR-2330-5p, bta-miR-339b, bta-miR-7864 |  |
| CSF2 |  | bta-miR-2295, bta-miR-2368-3p |  |  |
| CD14 | bta-miR-1249, bta-miR-219-3p, bta-miR-2326, bta-miR-329b, bta-miR-6120-5p, bta-miR-6531 | bta-let-7i, bta-miR-127, bta-miR-138, bta-miR-145, bta-miR-149-3p, bta-miR-185, bta-miR-2294, bta-miR-2309, bta-miR-2324, bta-miR-2375, bta-miR-2392, bta-miR-2449, bta-miR-2450a, bta-miR-2899, bta-miR-2904, bta-miR-34c, bta-miR-431, bta-miR-4657, bta-miR-502a, bta-miR-6519 |  |  |
| TLR-4 | bta-miR-1224, bta-miR-2320-5p, bta-miR-2338, bta-miR-2406 | bta-miR-15b, bta-miR-2316, bta-miR-2343, bta-miR-2888, bta-miR-2897, bta-miR-362-5p, bta-miR-375 | bta-miR-1225-3p, bta-miR-1260b, bta-miR-2334, bta-miR-2369, bta-miR-2388-3p, bta-miR-2392, bta-miR-2403, bta-miR-2425-5p, bta-miR-2450d, bta-miR-2461-3p, bta-miR-370, bta-miR-6535, bta-miR-7865 | bta-miR-2328-3p |
| LBP |  | bta-miR-2374, bta-miR-2450b, bta-miR-2474, bta-miR-2888, bta-miR-3141 | bta-miR-6535 |  |
| IL-10 |  | bta-miR-296-3p, bta-miR-1343-3p, bta-miR-2328-3p, bta-miR-2430 |  |  |
| TLR-2 |  | bta-miR-320a, bta-miR-23b-5p, bta-miR-2368-3p, bta-miR-2375, bta-miR-2426, bta-miR-2897, bta-miR-3602 | bta-miR-27a-5p, bta-miR-124a, bta-miR-124b, bta-miR-2422 |  |
| CCL-2 |  | bta-miR-2338, bta-miR-2349 | bta-miR-2309, bta-miR-2382-5p |  |
| MYD88 |  | bta-miR-296-3p, bta-miR-485, bta-miR-502a, bta-miR-2385-5p, bta-miR-2417, bta-miR-2466-5p, bta-miR-6525 |  |  |
| TNF-α | bta-miR-2389, bta-miR-2392 | bta-miR-2881, bta-miR-2460, bta-miR-6530, bta-miR-1287, bta-miR-2348, bta-miR-2357, bta-miR-2368-5p, bta-miR-320b, bta-miR-2447, bta-miR-2450c, bta-miR-4657 | bta-miR-329b, bta-miR-2452, bta-miR-3957 |  |
| CD83 |  | bta-miR-154b, bta-miR-1842, bta-miR-2375, bta-miR-2433, bta-miR-328 | bta-miR-2894 |  |
| C80 |  |  |  |  |
| IL-4 |  |  |  |  |
| IL-18 |  |  | bta-miR-21-3p, bta-miR-2308, bta-miR-2324, bta-miR-2328-3p, bta-miR-6534 |  |
| LY96 |  |  |  |  |
| ITGAM |  |  |  |  |
| IL-12A |  |  |  |  |
| MAPKAPK3 | bta-miR-103, bta-miR-27a-3p, bta-miR-107, bta-miR-34a, bta-miR-296-5p, bta-miR-328, bta-miR-485, bta-miR-1835, bta-miR-2309, bta-miR-2327, bta-miR-2387, bta-miR-2388-5p, bta-miR-2395, bta-miR-2449, bta-miR-1777b, bta-miR-2885, bta-miR-7862, bta-miR-1842 | bta-miR-26a, bta-miR-21-3p, bta-miR-145, bta-miR-199a-5p, bta-miR-205, bta-miR-27b, bta-miR-140, bta-miR-192, bta-miR-23a, bta-miR-425-3p, bta-miR-455-3p, bta-miR-532, bta-miR-23b-5p, bta-miR-365-5p, bta-miR-130b, bta-miR-188, bta-miR-196b, bta-miR-211, bta-miR-296-3p, bta-miR-329b, bta-miR-346, bta-miR-362-5p, bta-miR-432, bta-miR-449a, bta-miR-483, bta-miR-490, bta-miR-500, bta-miR-502b, bta-miR-504, bta-miR-631, bta-miR-671, bta-miR-877, bta-miR-940, bta-miR-1224, bta-miR-1225-3p, bta-miR-1282, bta-miR-1256, bta-miR-1251, bta-miR-1296, bta-miR-1193, bta-miR-1287, bta-miR-2287, bta-miR-2301, bta-miR-2313-5p, bta-miR-2324, bta-miR-2326, bta-miR-2328-3p, bta-miR-2330-5p, bta-miR-2331-5p, bta-miR-2339, bta-miR-2340, bta-miR-2346, bta-miR-2350, bta-miR-2356, bta-miR-2366, bta-miR-2378, bta-miR-2382-3p, bta-miR-2384, bta-miR-2385-5p, bta-miR-2389, bta-miR-2392, bta-miR-2394, bta-miR-2400, bta-miR-2402, bta-miR-2403, bta-miR-1584-5p, bta-miR-2411-5p, bta-miR-2411-3p, bta-miR-2415-5p, bta-miR-320b, bta-miR-2423, bta-miR-2425-5p, bta-miR-2438, bta-miR-2442, bta-miR-2454-5p, bta-miR-2454-3p, bta-miR-339b, bta-miR-2465, bta-miR-2469, bta-miR-2470, bta-miR-2471-3p, bta-miR-2473, bta-miR-664a, bta-miR-1388-3p, bta-miR-1468, bta-miR-2881, bta-miR-2882, bta-miR-2902, bta-miR-3578, bta-miR-1246, bta-miR-3141, bta-miR-378b, bta-miR-1247-3p, bta-miR-6528, bta-miR-6535, bta-miR-7857, bta-miR-2284ac, bta-miR-378d, bta-miR-4444 | bta-miR-345-5p, bta-miR-541, bta-miR-615, bta-miR-2342, bta-miR-2433, bta-miR-2464-3p, bta-miR-2467-3p, bta-miR-2901, bta-miR-3956, bta-miR-4449 | bta-miR-1777a, bta-miR-652, bta-miR-7863 |

CXCL-8 has bta-miR-2454-5p present in the 3 regions

TLR-4 has bta-miR-2328-3p present in the 3 regions

MAPKAPK3 has bta-miR-1777a, bta-miR-652, bta-miR-7863 present in the 3 regions
